# Food Allergy Prevention: Early Versus Late Introduction of Food Allergens in Children

**DOI:** 10.7759/cureus.21046

**Published:** 2022-01-09

**Authors:** Sandrine Kakieu Djossi, Anwar Khedr, Bandana Neupane, Ekaterina Proskuriakova, Keji Jada, Jihan A Mostafa

**Affiliations:** 1 Medical Research, California Institute of Behavioral Neurosciences & Psychology, Fairfield, USA; 2 Internal Medicine, California Institute of Behavioral Neurosciences & Psychology, Fairfield, USA; 3 Research, California Institute of Behavioral Neurosciences & Psychology, Fairfield, USA; 4 Psychiatry, California Institute of Behavioral Neurosciences & Psychology, Fairfield, USA

**Keywords:** food allergens, egg, fish, peanut, solid food introduction, allergenic foods exposure, allergy prevention in children, late introduction, early introduction, food allergy

## Abstract

The emergence of food allergies in children is crucial for various medical fields seeking a viable strategy for allergy prevention. The most well-recognized approach adopted by numerous health care and government institutions hinges on the delay in the introduction of food allergens, which supposedly protects infants from sensitization and decreases the possibility of allergy development. However, recent experimental findings indicate that the benefits of this approach might be overestimated, as early exposure to allergenic foods has been shown to yield more advantageous outcomes. Multiple investigations on the causes of allergic diseases report that avoiding food allergies might be related to early consumption of these allergens. Alternatively, delaying the contact with allergenic nourishments, explored in contemporary research, has been proven to result in a higher prevalence of allergies among children, originating such conditions as atopic diseases and extreme sensitization to foods. The current paper compares the two prominent strategies of allergenic food introduction, gathering the most pertinent modern evidence to distinguish whether exposure to food allergens should be delayed or advanced.

## Introduction and background

Food allergies among children and adolescents can have various consequences, ranging from minor food sensitivity to the risk of significant complications. Two methods of providing food allergens, namely early and late introduction, are often investigated as the most prominent strategies for preventing food allergies. While some scholars suggest that early exposure to food allergens can be more beneficial in the long term, others argue that late introduction is less dangerous for infants [1,2]. Delayed contact with allergenic materials such as eggs, milk, crustaceans, mollusks, fish, peanuts, tree nuts, soybeans, and wheat has been previously recognized as a leading technique to avoid the development of allergic or autoimmune responses [3]. It is worth noting that the recommendation was only based on experts' opinions as there were no convincing data to support this position at the time. Nevertheless, recent findings demonstrate that late introduction appears to be less efficient in preventing the emergence of food allergies than early allergen contact [4]. Considering the contradicting evidence, it is still unclear whether allergenic foods should be administered in the early or late growth periods to achieve the best possible results.

The trend regarding allergy prevention strategies has been primarily focused on the late introduction of food allergen in infancy with numerous recommendations from official sources, such as the American Academy of Pediatrics, promoting this strategy to accomplish the most productive protective effect [5]. However, contemporary observations from various countries state that late contact might be less efficient when contrasted with early exposure [6]. Furthermore, this strategy may even be ineffective in specific populations and cohorts, resulting in a higher prevalence of food allergies among children and adults [7]. In this regard, as the body of evidence supporting the early introduction approach continues to grow, the contradiction between the two techniques and their benefits for the children becomes more explicit. Therefore, additional studies are required to reduce the knowledge gap and establish the best method for allergy prevention. 

Promoting additional research and determining the most secure strategy for food allergy prevention can yield significant benefits for various populations. Given that the severity of food allergies can be a considerable health threat, especially with the emergence of anaphylactic shock, preventing any negative responses can substantially increase the welfare of affected individuals [8]. Ensuring that children safely consume and digest necessary nourishments is a pertinent issue, which could be resolved by establishing the best allergy prevention approach [9]. Furthermore, treating allergic reactions in adulthood is largely complicated and less effective [10]. Altogether, the current medical research must determine whether the early or late introduction of food allergens is the most beneficial for individuals in the long term.

The present review will discuss whether early introduction of food allergens is more effective in preventing food allergies than the late introduction of food allergens in children. According to current statistics, various food allergies have increased substantially in recent decades, and outgrowing allergic conditions is increasing more public health burden than in previous years [7,11,12]. Recently, such supplementary measures as improved research sponsorship and more accurate recommendations were adopted by some organizations [13]. However, even though some health institutions have updated their guidelines and changed their approach to allergy-related studies, the contradiction between early and late introduction remains crucial.

Defining risk and timing of the introduction of some allergenic food 

Infants are grouped high risk or low risk for food allergy development. High-risk infants have at least one parent or sibling with an allergenic condition such as food allergy, asthma, allergic rhinitis, or atopic dermatitis. Low-risk infants do not have the risk factor associated with high-risk infants [14].

The American Academy of Pediatrics in 2000 recommended delaying the introduction of allergenic foods like cow milk until one year of age and seafood or peanut until three years of age for infants at high risk of developing allergy. However, there is no evidence supporting the benefit of avoiding any solid foods beyond four months of age to prevent allergy [14].

## Review

Timing of food allergens and their outcome

Understanding the available methods of allergy prevention and exploring their aspects have been the focus of several studies. Authors from numerous countries conducted various types of research examining whether delayed exposure to food allergens can contribute to the low prevalence of food allergies in the affected populations and the effect of early introduction in infants [3,15,16]. For instance, Ierodiakonou et al. argue that the timing of allergenic food introduction is significant in hindering the development of allergic reactions to particular nourishments, specifically eggs and peanuts, common solid allergens [1]. According to the meta-analysis results, egg introduction at four to six months was associated with a lower risk of egg allergy than later egg introduction, while peanut introduction at age four to 11 months was also associated with a lower risk of peanut allergy [1]. However, early fish exposure was reported to have a low possibility of allergy prevention [17]. Therefore, although early intake of only egg and peanut allergens proved advantageous, this evidence demonstrates that certain foods should be introduced early to avoid future allergic complications. According to the benefits of late and early exposure, presented in Table 1, summaries highlight how the early introduction of various food allergens is associated with a decreased risk of developing food allergy and other allergies, while the later introduction does the reverse in children concerned.

**Table 1:** Description of selected meta-analysis and systematic review studies on the effects of early and late food allergens introduction

Study	Year of publication	Allergens examined	Effects of early introduction	Effects of late introduction
Ierodiakonou et al. [1]	2016	Egg, peanut	Lower risk of egg and peanut allergy	Increase the risk of egg and peanut allergy
Kattan [2]	2016	All common food allergens	Lessened possibility of sensitization, lessened risk of allergy to all common food allergens	Increased risk of food allergies and other allergic diseases
Tham et al. [18]	2018	All common food allergens	Positive effects for high-risk infants	Adverse manifestations of eczema for high-risk children
West [19]	2017	Peanut, egg, other food allergens	Diminished risk of developing food allergies, especially for high-risk infants	Increased risk of allergic reactions in high-risk children
Tham et al. [6]	2018	All common food allergens	Decreased risk of sensitization	Increased risk of sensitization
Burgess et al. [20]	2019	Egg, peanut, complementary solid foods	Risk of sensitization decreased, egg and peanut allergy development hindered	Increased risk of food sensitization, egg, and peanut allergy development

Primary investigations studies conducted by Johnson et al. and Perkin et al. and described in Table 2 show that introducing allergenic foods to infants between the ages of three to six months is achievable and might decrease the risk of developing food allergy in those children [4,21].

**Table 2:** Description of selected primary experimental studies on the safety and effects of timing of food allergens introduction

Author	Year of publication	Number of participants	Location	Allergens examined	Conclusion	Limitations
Perkin et al. [21]	2016	1,303	United Kingdom	Cow's milk, peanut, hen's egg, sesame, whitefish (cod), and wheat	Infants can safely be introduced to various food allergens before six months of age	Lack of generalization due to localized cohorts used
Jonsson et al. [4]	2017	65	Sweden	Complementary containing all common food allergens	Delaying the introduction of complementary foods, including various allergenic food, increases the risk of food allergies and other allergic disorders	Small sample of participants

Considering the outlined findings, this publication introduces a new question to be examined in further research. As the results regarding fish exposure were marked at low certainty, it is essential to identify the reasons behind such patterns and specify other foods that might demonstrate similar trends [4]. Nevertheless, such limitations as lack of relationship between food sensitization and allergic reactions and a small number of studies analyzed should also be noted [22,23]. Furthermore, no distinctions between cohorts were made in the evaluation, and some of the evidence used was retrieved from abstract articles.

The trends reported by Ierodiakonou et al. are highly consistent with other meta-analyses and systematic reviews identifying the efficiency of early egg and peanut allergens introduction [1]. Kattan, Tham et al., and Burgess et al. also provide sufficient evidence that ingesting egg and peanuts in periods four to 11 months can significantly lower the possibility of developing allergic reactions to these nourishments in the future [2,6,18,20]. West supports this proposition, presenting evidence that early introduction of peanut and egg allergens is dramatically more beneficial than delayed exposure [19]. Furthermore, high-risk infants and children manifesting signs of eczema and extreme sensitization are most positively influenced by early exposure [24,25]. Nevertheless, West notes that particularly early contact with any allergenic foods has been proven to raise gastrointestinal complications [19]. Thus, an optimal window for introducing food allergens should start at four to six months of age and should be concluded at 12 months, depending on the type of allergen.

Additional evidence regarding low-risk infants is presented in the study by West [19]. It appears that children from the general population can also be positively influenced by the early introduction of egg allergens, challenging previous suggestions that only high-risk infants may develop protection against allergies [19,26]. Nonetheless, the number of studies evaluated by West is considerably low, and the majority of them are focused on peanut and egg allergens [19]. In addition, the included articles were not consistently examined regarding their validity and the possibility of biased effects.

Similar to other mentioned articles, this investigation discusses enquiring about tolerance (EAT) research, which evaluated the relationship between low food diversity in early life and subsequent sensitization leading to the development of allergies. The EAT examination is numerously cited in various publications as primary evidence on the benefits of early allergenic food introduction and the demonstration of the multiple foods approach [27,28]. Indeed, the majority of contemporary studies focus on egg and peanut as the most prominent causes of allergies in children [29]. On the other hand, EAT explores other common allergens, namely cow's milk, peanut, hard-boiled egg, sesame, whitefish, and wheat [21]. The authors record that the prevalence of food allergies, in general, was significantly decreased after the early exposure to allergens, while delayed contact resulted in a 7.3% prevalence, which is a considerable gap [21]. Nonetheless, early and late introduction of other allergens, namely cow's milk, sesame, fish, and wheat, appeared to have no distinction in the development of either sensitization or allergies [30,31]. Therefore, the authors propose that less potent allergens are less dependent on the timing of exposure.

Apart from the aforementioned outcomes, the most beneficial finding of this study is the parental attitude toward allergenic food introduction. It was shown that there is another distinction between the early and delayed food consumption in infants, which is referred to as the predicament in feeding techniques [27]. However, the focus on parental impressions is one of the disadvantages of the research, which is extensively directed toward uncovering the mechanisms behind feeding complications. As only 42% of the overall sample were able to comply with the early introduction requirements, the study lost a remarkable portion of its credibility [19,32]. Although the authors note that they attempted to adjust the data according to the changes in the participant numbers, the derived conclusions should still be implemented carefully.

The examination of the fish allergen's early and late introduction in connection to allergy progression became the goal of one recent study. Jonsson et al. [4] investigated the infants who consumed this nourishment early or after a considerable delay. According to the discovered evidence, exposure to fish can be a significant factor in the development of allergic reactions to other solid foods and an allergy disease in general. The authors claim that nutrients contained in fish products are an essential element of protection against various allergic conditions, especially atopic diseases. Consuming food from an earlier age, such as four to five months, reduces the possibility of manifesting negative responses to various complementary foods, preventing further progression of allergies [33,34]. Therefore, although past studies have presented conflicting evidence concerning the benefits of early allergen intake, this research continues to investigate the significance of timely exposure to these foods.

It is essential to note that this research corroborates previously mentioned assumptions on the topic of negative effects following the late introduction of allergenic foods. While early ingestion of fish and eggs might prevent such ramifications as food allergies, asthma, and atopic diseases, delaying this contact can adversely affect children [35]. Information retrieved after the experimental intervention indicates that children who consumed allergens after 10 months of age were less protected against sensitization and allergy development, demonstrating a higher prevalence of allergies among this population [36]. This outcome has been observed not only in high-risk infants but also in standard-risk cohorts, strengthening the previously made propositions that all categories of young generations might benefit from early exposure to certain allergens.

Finally, an exceptional idea related to the attributes of allergy development is discussed in this study. Relying on past research, the authors note that high food diversity can be a prominent factor in the protection against allergic reactions in the future [37]. Although only a small sample of articles have conducted empirical investigations focused on the multiple foods approach, a tendency toward this method of experimentation can already be sighted [38-40]. In comparison with the delayed introduction of numerous foods, early exposure to a wide variety of complementary nourishments can significantly increase the strength of the autoimmune system, preventing food allergies in later periods [39,41]. Nonetheless, additional investigations are needed as Jonsson et al. only explore early and late introduction effects on a specific cohort, namely farm-raised and city-raised children [4]. Another drawback is the small sample size, meaning that the statistical power of uncovered impacts might be questionable.

Patterns of exposure to complementary nourishments

A pertinent complication in the development of food allergies is related to the introduction of other solid foods. Burgess et al. [20] evaluated the ingestion of such complementary foods as potatoes, vegetables, meat, rice, wheat, and cereal. Additionally, an investigation into classic categories of allergens, namely egg, peanut, tree nut, shellfish, fish, and sesame, was performed. Apart from highlighting the benefits of early egg and peanut introduction compared to delayed contact, the study revealed that early and late introduction of complementary foods does not increase the risk of food allergy [42]. However, evidence shows that consuming these products after four months appears to be related to a greater risk of food sensitization while ingesting them before four months is not [20,43]. Given that food sensitization might contribute to the development of allergy, this finding is crucial for understanding the relationship between solid foods introduction and allergic manifestations.

It is imperative to state the limitations of the studies performed. Although the statistical calculations and analyses were of high quality, a substantial number of the investigations included in the evaluation contained such problems as lack of population heterogeneity and differences between allergy risks [22]. Moreover, the standards of allergic reaction confirmation varied significantly, with only a minority of publications involving the standard oral food challenge [44]. Therefore, even though this article provides an insight into the development of food allergies after early and late exposure to complementary foods, the results of the investigation should be considered with caution.

Other specific food allergies are assessed in a recent systematic review focused on the rise in food allergy prevalence among the contemporary generations. Kattan claims that a recent increase in the likelihood of developing an allergic reaction to certain foods may indicate that the implemented methods are not as effective in battling this phenomenon, necessitating an improvement [2]. Another common type of allergenic food, milk, is evaluated in this study, introducing novel considerations regarding allergy introduction. According to the analysis, the timing of food introduction is highly crucial, and early exposure to milk can alleviate the negative consequences of an allergy progression [2,45,46]. However, given the small sample of included studies and limitations in the application of review strategies, evidence from this article should also be interpreted with additional caution.

Insights from Asian-focused research

While some studies report that Asian children obtain almost no protection from early introduction, others suggest that there is a distinct trend of sensitization for these individuals. According to Tham et al., allergy detection and prevention patterns vary substantially for Asian children, who demonstrate unique reactions toward the aforementioned strategies [18]. While for high-risk infants suffering from severe eczema and atopic dermatitis, a delayed introduction constitutes a considerable risk of developing an allergy in the future, and no credible data are available for medium-risk and low-risk children [6,47]. Furthermore, current investigations have shown that only peanut and egg allergens might negate the onset of allergic complications in Asian populations, and no findings have been reported considering other foods [48,49]. Therefore, with respect to the low prevalence of egg and peanut allergies in these ethnicities, supplementary examinations are required to establish a credible connection between the benefits of early and late introduction of food allergens. Currently, this approach appears to be advantageous only for high-risk infants.

The importance of the highlighted studies for the overall understanding of the discussed phenomenon is exceptionally high. An essential consideration is a novel perspective from Asian representatives, who are less susceptible to the most researched allergens. However, it is imperative to note that the investigations included in the study by Tham et al. were not examined for biases and contradictions, decreasing the quality of the conducted review and the presented findings [18]. Moreover, as only a small amount of evidence is available on this topic, the nature of the investigation remains exploratory, meaning that the results cannot be generalized and necessitate additional support from experimental research [50,51]. As for Tham et al., the conclusions of the statistical analysis appear contradictory, as only slight significance was found for the suggested correlations, and the number of participants involved in the study is low [6].

Limitations

The current review possesses several limitations to be considered. A prominent disadvantage is an underdeveloped body of research in this sphere, as additional evidence and data are required to establish a consistent pattern. After that, a significant number of discussed meta-analyses do not examine potential biases or experimental fallacies, thus decreasing the credibility of acquired information. Furthermore, as only a small portion of empirical investigations are included, this paper does not properly evaluate independent experimental data, relying on the summarized findings.

## Conclusions

To conclude, the two prominent strategies of food allergy prevention in children, namely early and late introduction to food allergens, were reviewed in detail in this article according to the recent academic literature. It appears that the timing of allergenic food consumption can tremendously impact the subsequent development of allergic reactions to various types of nourishments, from solid and complementary foods to specific allergens. The research evaluated has established that early exposure, between four to six months to primary allergenic foods, can be more efficient in preventing the emergence of food allergies in children and adolescents. In this regard, the early introduction of food allergens is especially beneficial for children. Compared with late consumption, it can remarkably strengthen the immune system, preventing adverse complications in the future. 

This review article provides a necessary insight into the problem of food allergy prevention in children and adolescents, which continues to rise substantially every year. Further investigations on this topic, especially the multiple foods approach, should be conducted. The adaptation of recent findings and the summary of the most significant articles in this area of investigation substantially benefit research on food allergy prevention. Finally, future studies can utilize the present review to provide an overview of contemporary approaches to food allergy prevention.
